# A systematic review of BCG vaccination policies among high-risk groups in low TB-burden countries: implications for vaccination strategy in Canadian indigenous communities

**DOI:** 10.1186/s12889-019-7868-9

**Published:** 2019-11-11

**Authors:** Lena Faust, Yoko Schreiber, Natalie Bocking

**Affiliations:** 1 Department of Epidemiology, Biostatistics and Occupational Health, McGill University, Montreal, Canada; 2 McGill International TB Centre, Montreal, Canada; 3 Section of Infectious Diseases, University of Manitoba, Winnipeg, Canada; 4 Clinical Sciences Division, Northern Ontario School of Medicine, Sioux Lookout, Canada; 5 Sioux Lookout First Nations Health Authority, Sioux Lookout, Canada

**Keywords:** BCG, Vaccination strategy, Tuberculosis, High-risk groups, Low-incidence countries

## Abstract

**Background:**

Bacille Calmette-Guérin (BCG) vaccination against tuberculosis (TB) is widespread in high-TB-burden countries, however, BCG vaccination policies in low-burden countries vary. Considering the uncertainties surrounding BCG efficacy and the lower likelihood of TB exposure in low-incidence countries, most have discontinued mass vaccination, choosing instead a targeted vaccination strategy among high-risk groups. Given the increased risk of TB infection in Canadian Indigenous communities compared to the general Canadian population, these communities are a pertinent example of high-incidence groups in an otherwise low-burden country, warranting particular consideration regarding BCG vaccination strategy. This systematic review aims to synthesise and critically appraise the literature on BCG vaccination strategies in high-risk groups in low-incidence settings to provide policy considerations relevant to the Canadian Indigenous context.

**Methods:** A literature search of the Medline and Embase databases was conducted, returning studies pertaining to BCG vaccine efficacy, TB incidence under specific vaccination policies, BCG-associated adverse events, and vaccination policy guidelines in low-burden countries. Study screening was tracked using the Covidence systematic review software (Veritas Health Innovation, Melbourne, Australia), and data pertaining to the above points of interest were extracted.

**Results:**

The final review included 49 studies, spanning 15 countries. Although almost all of these countries had implemented a form of mass or routine vaccination previously, 11 have since moved to targeted vaccination of selected risk groups, in most cases due to the low risk of infection among the general population and thus the high number of vaccinations needed to prevent one case in the context of low-incidence settings. Regarding identifying risk groups for targeted screening, community-based (rather than individual risk-factor-based) vaccination has been found to be beneficial in high-incidence communities within low-incidence countries, suggesting this approach may be beneficial in the Canadian Indigenous setting.

**Conclusions:**

Community-based vaccination of high-incidence communities may be beneficial in the Canadian Indigenous context, however, where BCG vaccination is implemented, delivery strategies and potential barriers to achieving adequate coverage in this setting should be considered. Where an existing vaccination program is discontinued, it is crucial that an effective TB surveillance system is in place, and that case-finding, screening, and diagnostic efforts are strengthened in order to ensure adequate TB control. This is particularly relevant in Canadian Indigenous and other remote or under-served communities, where barriers to surveillance, screening, and diagnosis persist.

## Background

Tuberculosis (TB) continues to be a significant global public health problem, with 1.6 million deaths being attributed to TB in 2017 [1]. Although implementation of the Bacille Calmette-Guérin (BCG) vaccine against TB is widespread in high-TB-burden countries, BCG vaccination policies in low-burden countries vary [2–5]. Although there is evidence for the efficacy of the vaccine for the prevention of miliary TB and TB meningitis in the pediatric population, other studies have highlighted limited protective effect [6]. In particular, this limited protective effect is highlighted in older age groups (as the vaccine prevents progression to active disease, but not infection with *Mycobacterium tuberculosis*) [6]. In addition, evidence for the duration of protection after vaccination has been varied, having been thought to last only approximately 10 years [7], although a study with a longer follow-up time of 60 years suggested continued substantial protection against primary TB disease over this period [8]. Further considerations regarding vaccination policy include the potential occurrence of adverse events, such as BCG-associated lymphadenitis and BCG-osteitis, and disseminated BCG infection in severely immune-compromised individuals [9, 10]. Limitations regarding the utility of the Tuberculin Skin Test (TST) as a screening tool for latent tuberculosis infection (LTBI) in widely BCG-vaccinated populations represent additional challenges [10].

The lower likelihood of TB exposure, the uncertainties surrounding BCG efficacy and length of protection, the potential occurrence of adverse events, and the higher number of vaccinations needed to prevent one case in low-incidence countries in comparison to high-incidence countries has rendered universal vaccination in most low-incidence countries unfavorable from a risk-benefit perspective [11–13]. Consequently, most low-incidence countries have discontinued mass vaccination of their general population, choosing instead a targeted vaccination strategy among high-risk groups [10, 13]. This shift to a targeted vaccination approach however comes with its own new challenges; the potential incomplete coverage of high-risk groups following the suspension of universal vaccination, due to discrepancies in adherence to policy, or difficulties identifying or reaching high-risk groups [14, 15].

Due to prevalent food insecurity, co-morbidities, and inadequate housing conditions, many Indigenous communities in Canada are at an increased risk of TB infection [16, 17], experiencing a TB incidence of 23.5/100,000 compared to only 4.8/100,000 in the general Canadian population [18]. This disparity in TB burden among the Canadian Indigenous population is rooted in the consequences of discrimination against Indigenous Peoples [16, 17]. The conditions experienced by Canada’s Indigenous communities during their forced assimilation on reserves and in residential schools facilitated the spread of infection, and exacerbated risk factors such as malnutrition [16, 17]. The Canadian Indigenous setting is therefore a pertinent example of a high-incidence community in an otherwise low TB burden country. This context therefore warrants particular consideration regarding TB control policy, including the determination of appropriate BCG vaccination strategies depending on the unique epidemiological profiles of different Indigenous communities.

The BCG vaccine was first introduced for use in Canadian Indigenous communities between the 1930s and 1950s [4]. However, following the BCG-associated deaths of six immune-deficient First Nations children and the occurrence of other serious adverse events as a result of BCG vaccination, it was recommended in 2006 by the National Advisory Committee on Immunization that routine BCG vaccination be discontinued among First Nations and Inuit communities [19, 20]. Routine BCG vaccination was instead to be replaced by targeted vaccination in communities with an incidence of smear-positive pulmonary TB ≥15/100,000 population in the last 3 years, with an annual risk of TB infection ≥0.1%, or with limited diagnostic services [20]. Replacing routine with targeted vaccination however brings with it challenges in terms of appropriately identifying high-risk groups and ensuring adequate coverage among them.

Given the varied historical context of BCG vaccination in Canadian Indigenous communities, the persisting uncertainty regarding its benefit in low-burden settings, and the ongoing question of how to optimally define and reach high-risk groups within these low-burden settings, this report aims to systematically review the findings of previous studies regarding BCG vaccination policy in low-burden settings. In particular, results pertaining to vaccine efficacy, occurrence of adverse events, implications of vaccine withdrawal, and overall vaccination policy recommendations are reviewed, in order to provide an evidence base for BCG vaccination programming in the Canadian Indigenous community context.

## Methods

### Search strategy

A literature search of the Medline and Embase databases was conducted in January 2018, using the detailed search strategy shown in Additional file 1**.** The search was filtered to return only studies published after 1988, and written in English or French. Studies pertaining to BCG vaccine efficacy, TB incidence under specific vaccination policies, or BCG-associated adverse events, as well as general BCG vaccination policy guidelines in low-burden countries were included. Detailed inclusion and exclusion criteria regarding publication time frame, language, study population, study design, and reported outcomes are outlined in Table 1**.** The methodology of this review is presented according to the PRISMA guidelines for the reporting of systematic reviews and meta-analyses [24].

**Table 1:** Study Inclusion and Exclusion Criteria

Inclusion Criteria	Exclusion Criteria
Publication Timeframe and Language	
• Studies published after 1988 • Studies published in English or French	• Studies published before 1988 • Studies in languages other than English or French
Study Population	
• Taking place among a high-risk group in a low TB-burden country o Low-burden countries are defined as those with less than 100 TB cases per million population [21].	• Not taking place among a high-risk group in a low TB-burden country (or, if including both low and high-burden countries, not stratifying results by burden level). o Countries excluded in the initial search were those on the current list of high-TB-burden countries provided by the WHO [22] (see Additional file 1). o Further countries were excluded during abstract screening if they did not meet the WHO’s low incidence cut-off of less than 100 TB cases per million population after consultation of the WHO TB database [23]. • Studies taking place in other contexts considered not relevant to BCG vaccination considerations in the Canadian Indigenous setting, (e.g. studies taking place only among immune-compromised subjects)
Study Design	
• Epidemiological studies • Case studies • Public health policy reviews or reports	• Animal, ex-vivo, molecular biology or non-clinical studies • Editorials, commentaries, conference abstracts, or outdated policy reports for countries for which newer versions were already included in the review
Reported Outcomes	
• Studies providing information on BCG vaccination policies, and specifically, information regarding: o Effectiveness of BCG in the target population o Possible harms of vaccination o Possible harms / implications of discontinuation of vaccination	• Studies not relating to BCG vaccination for TB (e.g. studies on TB screening or diagnosis) • Studies on novel vaccine development • Studies not providing information on BCG vaccination policies, and specifically, information regarding: o Effectiveness of BCG in the target population o Possible harms of vaccination o Possible harms / implications of discontinuation of vaccination • Studies on vaccination policy of healthcare personnel

(The review protocol was not registered on PROSPERO).

### Data collection and synthesis

**Fig. 1:**
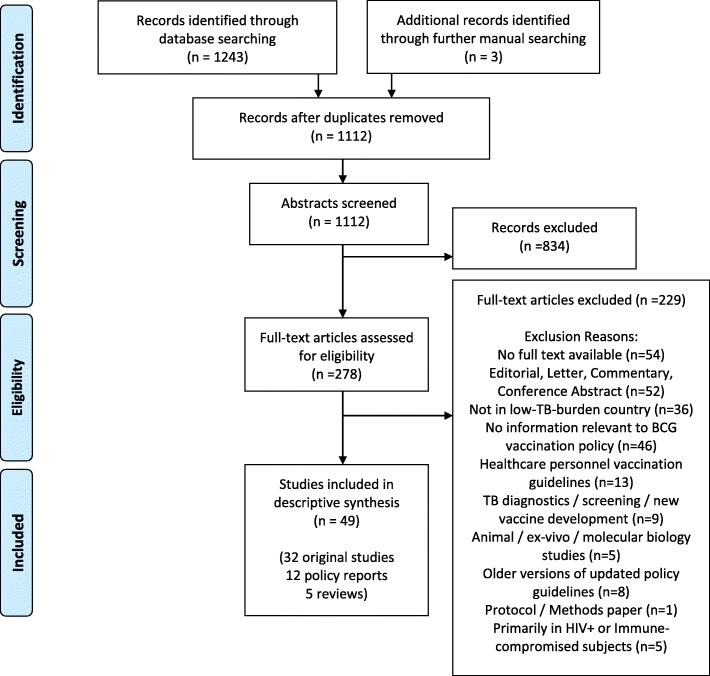
Search and Screening Process (PRISMA Flowchart)

The initial search returned 1109 abstracts after duplicate removal, and three additional studies were included via manual searches. The 1112 records were then screened and evaluated against the above inclusion criteria. Following 834 exclusions, full texts for the remaining 278 records were retrieved and read. Following full text review of 278 articles, 229 records were excluded (For reasons such as: not taking place in a low-burden country (*n* = 36), not providing information relevant to BCG vaccination policy (*n* = 46) (e.g. assessing healthcare provider’s knowledge of policy), or being editorials, commentaries, letters or conference abstracts (*n* = 52)), leaving 49 studies remaining in the final review. Of these, 32 were primary (original research) studies, 12 were policy reports, and 5 were reviews. Figure 1 shows the study screening process and provides detailed exclusion reasons. Study de-duplication, screening, and inclusion was tracked using the Covidence systematic review software (Veritas Health Innovation, Melbourne, Australia), and relevant data regarding vaccine efficacy, adverse events, future policy recommendations and implications of withdrawal were extracted in Microsoft Excel (version 14.5.5).

As studies reported varying TB incidence data and BCG vaccination policies depending on publication date, for the policy overview, national TB incidence per 100,000 population per year was calculated for each country included in the review, based on the 2016 incidence reported in the World Health Organization Tuberculosis country profile database [23], and current BCG vaccination policies were extracted from the recently updated BCG World Atlas [4]. On the other hand, information regarding vaccination policy and associated outcomes regarding TB incidence and adverse events over particular time periods was extracted from each specific included study. Based on these results, recommendations are made regarding BCG vaccination policy in the Canadian Indigenous context, specifically in Canadian Indigenous communities.

### Risk of Bias assessment

The methodological quality of the 32 primary research studies included was assessed at the study level using risk of bias assessment tools appropriate to the corresponding study design. RCTs (*n* = 4) were assessed using the National Heart, Lung and Blood Institute’s (NHLBI) Quality Assessment Tool for Controlled Intervention Studies [25], case-control or case-cohort studies (*n* = 3), were evaluated using the NHLBI’s Quality Assessment Tool for Case-control Studies [25], quasi-experimental studies (*n* = 5) were evaluated via the Joanna Brigg’s Institute (JBI) Critical Appraisal Checklist for Quasi-Experimental Studies [26], and cross-sectional or observational cohort studies (*n* = 15) were assessed using the NHLBI’s Quality Assessment Tool for Observational Cohort and Cross-Sectional Studies [25]. Studies are classified as having low, unclear or high risk of bias for each of the quality criteria assessed and the results presented graphically. The case report was descriptively evaluated using the JBI Critical Appraisal Checklist for Case Reports [26], and modelling studies (*n* = 4) were assessed based on the ISPOR Principles of Good Practice for Decision Analytic Modelling in Health-Care Evaluation [27], scored depending on adherence to these principles.

## Results

A PRISMA [24] diagram of the screening results is shown in Fig. 1**.**

### Study characteristics

Of the 49 included studies, 32 were primary (original research) studies, 12 were policy reports, and 5 were reviews. The primary studies had a variety of study designs, including RCTs (*n* = 4), case-control or case-cohort studies (*n* = 3), quasi-experimental studies (*n* = 5), cross-sectional studies (*n* = 8), retrospective or prospective cohort or observational studies (*n* = 7), modelling (simulation) studies (*n* = 4), and one case report.

Among the 12 policy reports and 32 primary studies, in terms of countries of conduct (all of which must have had a TB incidence rate of < 100 cases per million population as a criterion for inclusion), most took place in European countries (France *n* = 6, UK *n* = 6, Denmark *n* = 4, Sweden *n* = 3, Netherlands *n* = 2, Finland *n* = 2, Norway *n* = 2, Czech Republic *n* = 2, and Ireland *n* = 1). Further studies were conducted in Canada (*n* = 3), the USA (*n* = 3), Australia (*n* = 3), Saudi Arabia (*n* = 1), the United Arab Emirates (*n* = 1), and Egypt (*n* = 1). The remaining 4 studies spanned multiple countries (with one including the USA, Canada and Greenland^1^), whilst others provided information on BCG vaccination across low-incidence countries in general. The 5 reviews focused either on the UK (*n* = 1), France (*n* = 1), or general low-incidence settings (*n* = 3).

Of the 32 primary studies, 16 reported on vaccine efficacy or TB incidence under different BCG vaccination policies, 15 reported on adverse events associated with BCG use, or provided other recommendations regarding vaccination policy (14 studies and one case report), and 1 study reported both [10].

### Overview of vaccination policies by TB incidence in included study countries

A summary of policy recommendations in included study countries, by national TB incidence, is provided in Table 2**.** As the included studies report different policies and incidence rates depending on publication date, current policies in this summary table are extracted from the BCG World Atlas [4], which was last updated 2017, unless otherwise indicated. National incidence rates per 100, 000 population for each country were calculated based on 2016 WHO incidence estimates, whereas incidence rates in specific targeted risk groups (where available) as well as policy recommendations based on study observations are taken from individual studies included in the current review.

**Table 2:** Overview of Vaccination Policies in Countries of Conduct of Included Studies, by TB Incidence

Country	#S	Prior Vaccination Policies	Current Vaccination Policy ^b^	As of (Year)	Age at Vaccination	Currently Targeted Risk Groups (TRG)	Nat. TB Inc. ^a c^	TB Inc. ^a^ in TRG	Summary of Policy Recommendations or Concerns Based on Results of the Included Studies (see Tables 3 and 4 for specific results regarding vaccine efficacy and adverse events)
UAE ^d^	1	Mass vaccination	No change - Mass vaccination	NA	At birth	NA	0.7	NA	Low number of adverse events suggests vaccine is safe and current policy of mass vaccination is acceptable [28].
USA ^d^	3	Targeted	No change - Targeted	NA	NR	Children residing in contexts in which risk of TB infection is high (detailed cut-offs unspecified)	2.9	NR	Mass vaccination remains unnecessary [29]. Regarding high-risk groups, BCG vaccination among the homeless has been shown to be effective [30], and the protective efficacy of the vaccine among Native American communities has been shown to persist up to 50–60 years post-vaccination, suggesting a long-term benefit of vaccination among this risk group [8].
Finland	2	Mass vaccination	Targeted	2006	At birth (and previously, re-vaccination at school age)	• Children of immigrant families from high-incidence countries, • Children living with relatives with TB • Children staying in high-incidence countries for a prolonged period • Children whose families request BCG • Children from high-incidence countries who have not yet entered school.	4.3	NR	In Finland, most TB cases are among seniors (65 or older) and the Indigenous population (rather than among immigrants, as is the case in many other European countries). However, recommended target groups remain immigrant and other at-risk children (see targeted risk groups) [31]. Before routine vaccination was discontinued in 2004, it was emphasized that the following criteria (set by the International union against TB and lung disease) should be met before discontinuation: • Strong established TB control program • Knowledge of impact of HIV prevalence in the population on TB transmission • Incidence of smear-positive TB no more than 5 / 100,000 in the past 3 years, or • Less than 1 case per 10,000,000 of TB meningitis in children under 5 in the last 5 years, or • Annual risk of TB 0.1% or less [32].
Czech Republic	2	Mass vaccination	Targeted	2010	Between birth and 6 weeks of age (and previously, re-vaccination at 11 years)	Newborns from high-risk families	4.7	NR	Although TB incidence increased in the region where mass vaccination was stopped [10], risk of infection was low, so re-introduction of mass vaccination was not deemed necessary [11]. Overcrowded living conditions were associated with a higher risk of infection, which is also a relevant consideration in the Canadian Indigenous context, however, no specific risk groups or criteria for targeted vaccination were identified, except neonates from families at “high risk” of infection [11].
Canada	3	Routine vaccination among Indigenous communities	Targeted (see currently targeted risk groups)	2003–2005 (Varies by community)	Within 1 year of birth	• Infants in some Indigenous Communities, • Infants in populations with an annual risk of TB infection > 0.1%	4.8	26.6	As recommended by the National Advisory Committee on Immunizations, vaccination should continue in: • Infants in First Nations and Inuit communities with an average annual rate of smear-positive pulmonary TB greater than 15 per 100,000 population in the past 3 years, or • Infants residing in populations with an annual risk of TB infection greater than 0.1% [33]. Where vaccination is withdrawn, it should be ascertained that a strong surveillance system is in place before withdrawal [19].
Netherlands	2	Targeted vaccination of children with parents from endemic countries	Targeted	2005	NR	Children with one or both parents born in a country with TB incidence > 50/100000 (as per WHO estimate)	5.2	NR	Issues of incomplete coverage (39% of at-risk children not vaccinated in a 2014 study) suggest that further cases could be prevented through stricter adherence to vaccination of high-risk children. Specifically, the continuation of targeted vaccination in new-borns of parents from TB endemic countries (WHO-estimated incidence > 50 /100.000 population) is recommended [14].
Norway ^d^	2	Mass vaccination	Targeted	2009	At birth	Newborns with parents from high prevalence countries	5.6	NR	Targeted vaccination of high-risk groups recommended over universal vaccination due to high number of vaccinations needed to prevent one case in low-risk groups [34]. Due to concerns of incomplete coverage of risk groups after discontinuation of universal vaccination policy, improvements are needed in the identification of children considered high risk [35].
Australia	3	Mass vaccination	Targeted	Mid-1980s	Previously at school age, currently at birth	• Newborns in high-incidence Aboriginal and Torres Strait Islander communities • Newborns in families with leprosy, • Newborns and children otherwise at risk (e.g. those who will be travelling to high TB prevalence countries	5.7	Foreign-born pop.: 15.5–21.0 (1992–2012) Aboriginal pop.: 5.9 (2008)	As most TB cases (80–90%) in the country are among immigrants from high-prevalence countries, selective vaccination is efficient [36]. Increased surveillance of adverse events is needed during vaccine shortages and consequent use of unregistered vaccines [37, 38].
Denmark	4	Mass vaccination	Targeted	1986	At birth	• Children traveling to endemic countries • Children with a family history of TB	5.8	NR	Targeted vaccination continues to be recommended. No significant negative effects of BCG were found on child psychomotor development [39], occurrence of inflammatory bowel disease [40], or other adverse events [41].
Ireland ^d^	1	Mass vaccination (in some regions)	No change - Mass vaccination in some regions	NA	At birth (and previously, re-vaccination at 11–12 years)	NA	6.8	NA	A 1997 study in Ireland showed the number of vaccinations needed to prevent one case had decreased over time, suggesting that continuing the policy of routine neonatal BCG vaccination is beneficial [42], however, this policy may need to be updated based on the current situation.
Sweden	3	Mass vaccination	Targeted	1975	At 6 months - 1 year (and previously, re-vaccination at 7 years)	High risk infants, i.e. those with: • Family history of TB, • Close contact to TB case • Parents from endemic countries • Plans to travel to endemic countries [43]	7.4	NR	Targeted vaccination of high-risk groups should continue, however, the age at vaccination should be postponed from at birth to 6 months of age, to allow the detection of immune-compromising conditions prior to vaccination (thereby avoiding serious adverse events) [44].
France	6	Mass vaccination	Targeted	2007	Before hospital discharge or entry into daycare	• Children with parents from endemic countries • Children with a family history of TB	7.6	NR	No significant difference in incidence of TB meningitis before and after the changes in vaccination policy, suggesting that targeted screening is sufficient for TB control. However, given the possibility of incomplete coverage of high-risk children after suspension of mass vaccination, surveillance efforts should be strengthened [12].
Egypt ^d^	1	Mass vaccination	No change - Mass vaccination	NA	At birth	NA	8.6	NA	Routine vaccination at birth continues to be considered beneficial (based on the significant correlation of BCG vaccine coverage with reduced TB incidence and TB-associated mortality) [45].
Saudi Arabia ^d^	1	Mass vaccination	No change - Mass vaccination	NA	At birth	NA	9.3	NA	Routine vaccination continues, however, given the change of strains used for vaccination (from Pasteur 1173 P2 and Tokyo 172–1 to the Danish 1331 strain in 2005), more detailed population-based studies are recommended before the introduction of new strains in the future, with particular attention to the prevalence of host risk factors that contraindicate vaccination, such as immunodeficiency [9].
UK	6	Mass vaccination	Targeted	2005	At birth in at-risk neonates and opportunistically in older children [46] (previously at 12–13 years)	Children from high-risk families (see recommendations for details)	9.4	NR	Re-introduction of routine vaccination is not recommended [13]. Instead, targeted vaccination is recommended among the following groups: • Infants attending primary care organisations with a high incidence of TB, • Infants in low-incidence communities meeting the following criteria:– those born in an area with a high incidence of TB, or– those with one or more parents or grandparents who were born in a high-incidence country (> 40 cases per 100,000 per year), or– those with a family history of TB in the past 5 years. • Children younger than 16 who also meet these risk criteria should be opportunistically vaccinated [47].

*#S* Number of included studies from this country in this review, *Cont* Control, *Inc* Incidence, *Int* Intervention (or case group, for case-control studies), *NA* Not applicable, *Nat* National, *NR* Not reported, *Pop* population, *TB* Tuberculosis, *TRG* Targeted Risk Groups

^a^ Cases per 100,000 population per year

^b^ As the included studies report different policies as current or prior depending on publication date (as shown in later tables), current policies in this summary table are extracted from the BCG World Atlas, which was last updated 2017, unless otherwise indicated

^c^ National incidence rates per 100, 000 population were calculated based on 2016 WHO incidence estimates

^d^ Policy information last updated 2011

As shown in Table 2, incidence rates in the included study countries ranged from 0.7/100,000 population/year (in the UAE) to 9.8/100,000 population/year (in the UK) (as per the review’s exclusion criteria, only countries with incidences < 10/100,000 (i.e. < 100/1,000,000) are included). Although almost all of the 15 countries had at some point implemented a form of mass or routine vaccination previously, mass vaccination continues in only 4 of these countries today (Egypt, Saudi Arabia, the UAE, and, in some regions, Ireland). All 11 remaining countries have since moved to targeted vaccination of selected risk groups, instead of universal vaccination. It is interesting to note that whilst three of the four countries continuing mass vaccination have relatively high incidence rates in comparison to the other low-burden countries (Saudi Arabia: 9.3/100,000 Egypt: 8.6/100,000 Ireland: 6.8/100,000 population/year), the fourth, the UAE, continues mass vaccination despite having the lowest TB incidence of all 15 countries (0.7/100,000 population/year).

In countries currently implementing targeted vaccination, risk groups identified for targeting most often included children with parents from TB endemic countries, or children with a family history of TB or contact with a TB case, who had a non-reactive TST. Common reasons cited for the shift from a universal to a targeted vaccination program were the low risk of infection among the general population and thus the high number of vaccinations needed to prevent one case in the context of low-incidence settings (for example, an estimated 21,699–25,125 vaccinations needed among the Norwegian adolescent population to prevent one case) [11, 34], and the concentration of the majority of cases among a specific risk group [36]. In countries where mass vaccination was deemed to still be justified, reasons for continuing mass vaccination included the low incidence of serious adverse events associated with the vaccine [28], and findings regarding a significant correlation of BCG vaccine coverage with reduced TB incidence and TB-associated mortality rates (in Egypt) [45].

Notably, among studies in countries that have shifted from universal to targeted vaccination, all found this change in policy appropriate and did not recommend re-introduction of universal vaccination, however, possible dangers of withdrawal of mass vaccination were also highlighted. These included not only a potential rise in TB cases, (as occurred, for example, in the Czech Republic, where a rise in TB incidence was observed in the region in which mass vaccination was discontinued in 1986 [9]), but also the concern of incomplete coverage of risk groups after discontinuation of universal vaccination [35]. It was therefore generally agreed that for successful withdrawal of universal vaccination, effective identification of high-risk children and strict adherence to guidelines regarding their vaccination is needed [14, 35], and a strong TB control program (including sufficient screening and diagnostic strategies) must be in place prior to withdrawal [19, 32].

### BCG vaccine efficacy and effect of vaccination policies on TB incidence

Study findings regarding vaccine efficacy and the effect of changes in vaccination policies on TB incidence are shown in Table 3 (grouped by country). Although two-arm studies comparing vaccinated to non-vaccinated groups (or areas with higher vs. lower vaccine coverage) generally report a higher incidence of TB in non-vaccinated (or low-coverage) groups compared to vaccinated (or high-coverage) groups [8, 30, 42, 55, 56]. Despite this, reported vaccine efficacy however varied widely across studies, ranging from 49% (95%CI: 14–62%) in a study of Asian children living in the UK [53] to as high as 87.5% (95%CI: 30–98%) in a study of the general French population [50]. Findings that supported targeted vaccination among selected risk groups rather than universal vaccination included a high number of vaccinations needed to prevent one TB case in low-burden areas [34], along with a potentially high number of adverse events per prevented case [52], and the persisting low risk of infection in low-incidence settings making mass vaccination unnecessary [10, 11, 13]. It was also emphasized that a further benefit of the discontinuation of mass vaccination in low-incidence settings was the restoration of the diagnostic utility of the TST for the identification of LTBI [10]; an important consideration given that detection and prophylaxis of LTBI continues to be a component of TB control in the Canadian Indigenous context [57], and because interferon-γ-release assays are not widely available [58].

**Table 3:** Primary Outcomes: TB Incidence and Vaccine Efficacy under the Evaluated Prior and Current Vaccination Policies, by Country

Study Characteristics										Study Outcomes			Policy Recommendations	
Ref, Year	CTR	ST	Study Pop	Current / prior BCG policy (cov) ^a^	New policy (cov) ^a^	Int description ^b^	Cont description	N (int)	N (cont)	TB Incidence and Vaccine Efficacy			Risk groups for Targeted Vaccination	Observations and General Recommendations
										TB Inc. (cont) n/N	TB Inc. (int) n/N	% Efficacy (95%CI)		
[48], 1990	Can	CC	Alberta indigenous communities (Treaty Indians)	All Treaty Indians in Alberta are given BCG vaccination at birth since 1948		Cases - TB	No TB	160	314			57 (23.4–75.3)	Indigenous population	Vaccination in this population should continue until incidence rates fall (cut-off not specified) (as benefits in this high-incidence community outweigh risks and costs), however, vaccination at an older age (instead of at birth) warrants consideration given the finding of higher protection among those vaccinated after 6 months of age (63% efficacy after 6 months of age, 42% before 6 months). Diagnosis and treatment should be strengthened as control measures.
[11], 1994	Czh	CS	Non-BCG vaccinated children	Mass BCG vaccination in all newborns (discontinued in 3 regions of the country in 1986)	Vaccination only in high-risk infants	Non-vaccinated	NA	184,648	NA	LTBI: 283/184648			Unspecified high-risk infants	Risk of infection was low, so re-introduction of mass vaccination is not necessary. Overcrowded living conditions were associated with a higher risk of infection (also relevant to Canadian Indigenous context).
[10], 1993	Czh	QE	Czech children	Mass vaccination	Discontinuation of mass vaccination in one region in 1986	Region of ceased vaccination	Regions of continued mass vaccination	148,560	600,195	31/148560 (not vaccinated)	24/600195 (vaccinated)	80%		Although incidence increased in the region where mass vaccination was stopped, mass vaccination found to be obsolete due to the small number of children that were not vaccinated developing TB. The cessation of mass vaccination is additionally beneficial due to the restoration of the utility of the TST as a screening tool. Implications of Withdrawal: 25.6 cases could have been prevented through continuation of mass vaccination in the region where it was stopped.
[12], 2015	Fr	CS	French Children	Mass vaccination until 2007	Vaccination in high risk groups	Children in Ile-de-France (all vaccinated, as all considered high risk)	Children in other regions of France where mass vaccination was ceased			Regions where only at-risk children are vaccinated: 2000–2005:0.30, 2006–2011: 0.47	Ile-de-France (all considered at risk, all vaccinated): 2000–2005: 1.14, 2006–2011: 0.29 per million			There was no significant difference in incidence of TB meningitis before and after the changes in policy, suggesting that targeted screening is sufficient for TB control, however, given the possibility of incomplete coverage of high-risk children after suspension of mass vaccination, surveillance efforts should continue.
[49], 2011	Fr	QE	General French population	Universal BCG vaccination (47%)	Lower coverage due to de-commercialisation of vaccine	2008 coverage (lower)	2006 vaccination coverage (higher)			82 cases (in children under 3)	105 cases (in children under 3)			Implications of withdrawal: Increase in number of cases reported in 2008 (low vaccine coverage) compared to 2006, where vaccine coverage was higher - although it is unclear whether this change indicates an actual increase in transmission, or rather is the result of improved surveillance
[50], 1994	Fr	RC	General French population	Routine vaccination at birth (80% in children under 5 in 1990)						70 cases of TB meningitis in total population (1.2 per million inhabitants (95% CI: 0.9–1.5), 6 in under 5 age group		87.5% (30–98)		Routine BCG vaccination continues to play an important role in TB control in France, and should be maintained unless the risk of infection continuous to reduce.
[42], 1997	Ire	QE	Children (15 or younger) in Ireland	Dependent on area (see int/cont description) (38% in 1986 to 35% in 1991)		Areas with neonatal BCG vaccination	Areas without neonatal BCG vaccination	children < 15, 1986 and 91: 751813, 680,269	269,350, 282,489	1986: 14.1/100000, 1991: 13.4/100000	1986: 4.9/100000, 1991: 3.4/100000	In areas with vs. without neonatal BCG vaccination: IRR = 1.92 (95% CI = 1.47–2.40) *p* = 1.5 × 10–5 in 1986; and IRR = 2.12 (95% CI 1.75–2.58), *p* = 1.0 × 10–7 in 1991	Neonates	TB incidence in areas without a policy of neonatal BCG was significantly higher than areas where neonates were vaccinated. As the number of vaccinations needed to prevent one case decreased over time (1986: 646, 1991: 551), this suggests that continuing the policy of neonatal BCG vaccination is beneficial. Also, based on the WHO’s recommendations that routine vaccination of newborns should not be continued until: • The average incidence is < 5 cases per 100,000 per year in three successive years, − • The annual risk of tuberculosis infection is ≤0.1%, • A 10% decrease in tuberculosis annually for a period of 10 years is observed • Or the incidence of childhood tuberculosis meningitis is < 1 case per 10 million per year which was not yet the case in Ireland at the time of the study.
[51], 2009	LIC	M	General population in LIC										A common reason for the discontinuation of mass vaccination programs has been the trade-off between vaccinating and being able to effectively detect LTBI, with most low-incidence countries having suspended mass vaccination and focusing instead on the accurate identification and subsequent prophylaxis of LTBI, however, according to this model, low-incidence countries do not detect and treat LTBI at rates high enough to benefit from the cessation of mass vaccination programs. (Note that the model assumed that LTBI in previously vaccinated individuals is completely undetectable, regardless of time since vaccination, and thus represents a conservative estimate of the benefit of mass vaccination). Based on US life expectancy, incidence and cure rate data, mass vaccination begins to become beneficial at a vaccine efficacy of 50%. (Given that many of the studies included in the current review report higher efficacies in their populations, this modeling study suggests that mass vaccination continues to be beneficial in a low-incidence country context).	
[52], 2008	LIIC	M	Children in LIIC										Universal vaccination was found to be beneficial in settings with a prevalence of approx. 30 sputum smear positive cases per 100,000. If prevalence is below 5 per 100,000, a universal vaccination strategy may not be beneficial due to high incidence of adverse events per case prevented. Note that the model assumes 100% BCG coverage and a vaccine efficacy of 80% against both meningitis and miliaryTB.	
[34], 2009	Nor	QE	Native-born (non-immigrant) population < 30 years old	Vaccinates all 12–14 year olds.	NA	Vaccination policies in Sweden, Finland and Denmark	Norwegian vaccination policy	Py: Nor: 16, 567, 951	Py: Swe: 29, 851, 794 Den: 18, 042, 696 Fin: 18, 353, 224	Per100 000 py: (95%CI) Nor: 0.45 (0.36–0.57)	Swe: 0.56 (0.48–0.65) Den 1.41 (1.24–1.59) Fin: 0.65 (0.54–0.78) IRR: Fin vs. Swe: 0.45 (95%CI 0.22–0.94)	61–64 (Norwegian 15–29-year-olds) 67–71 (after adjustment for coverage) Average annual # of prevented cases 1.9–2.2.	Children with origins in high-incidence countries or travelling to high-incidence countries	Protective effect of BCG vaccination of newborns in Finland, and adolescent vaccination in Norway among persons at low risk of TB, however, a high number of vaccinations (21699–25,125) were needed to prevent one TB case among the low-risk population. The Norwegian Ministry of Health therefore decided in 2009 to discontinue vaccination among low-risk groups but continue it among high-risk groups.
[43], 2006	Swe	RC	Swedish population	Routine vaccination of newborns until 1975 (discontinued due to high incidence of bcg osteitis (more than 95% before 1975)	Targeted, in high-risk groups < 2% in 1976–1980, and 16% post 1980. (88% of targeted risk groups))	Born before 1974 (routine vaccination)	Born after 1974 (targeted vaccination)			For the birth cohort born in 1975 and followed up to 2004: 0.5/100,000py (227 cases) (6.4 per 100,000 in 2005)		85% in 1969–74	High risk infants include those with - Family history of TB, - Close contact of TB case- Origin from endemic countries (whether born in Sweden or not), − Planned travel to endemic countries	An increased incidence of TB in the mostly non-BCG vaccinated cohorts born after 1975 was observed vs those born during routine vaccination period (1969 to 1974). In addition, the recommended age at vaccination was recommended to be increased from at birth to 6 months in 1994, to avoid vaccinating newborns who may later develop immune-deficiencies.
[13], 1991	UK	RC	Population of Oxfordshire, UK	Routine vaccination of 13 year olds in schools, until 1981	Cessation of routine vaccination					4 cases since 1981, of children who had not been routinely vaccinated at school. All four had other risk factors apart from being unvaccinated.			Neonates of African, Asian, Central and South American and Middle Eastern origin, those with a family history of tuberculosis, contacts of TB cases, children newly immigrating from or travelling to endemic countries.	Given the continued decline in cases from 1973 to 89, (94 vs. 24 cases), does not recommend re-introduction of universal vaccination, but seeing as the 4 cases all had other risk factors, recommends improvement in identification and subsequent vaccination of at-risk groups
[53], 1991	UK	CC	Asian Children living in England	(51 and 64% in cases and controls, respectively)		Cases - TB	No TB	111	555			49 (14–62)	Children originating in endemic countries (or whose parents originate in these countries)	Continued vaccination of high-risk children, e.g. those originating in endemic countries. As the incidence in this group is decreasing, this policy may be reviewed in the future.
[54], 1989	UK	M	UK general population	Universal vaccination through school programs at age 14 (75–80%)										Gradual increase in vaccinations needed to prevent one case: 5800 in 1994, 9300 in 1999 (as risk of infection also decreased: 1:17000 to 1:26000). Implications of withdrawal: If universal vaccination were stopped in 1991, case notifications would increase by 80, and 50 if stopped in 1996. (These new infections would mainly be in the 15–29 year old age group and would comprise existing as well as new sources of infection in the community)
[55], 2018	USA, Can, Grn	PC	Canadian, American and Greenland Indigenous populations	Mass vaccination of infants in some study years, but discontinued in others. (Greenland, Eeyou Istchee, Nunavik, and Nunavut: at least 80%; First Nations population of Alberta: 50–60%)						Decrease in annual TB incidence attributed to population-based BCG vaccination: −10% (95% CI −5 to −15) (adjusted for infant mortality and crowded housing)			Indigenous populations	Population-based vaccination was significantly associated with a decrease in TB incidence. This remained true when adjustment was made for improvements in overall health and socioeconomic status over the years of the study. This therefore suggests that population-level vaccination in high-risk groups (rather than vaccination based on individual risk factors) may be beneficial in the Indigenous community context. Implications of withdrawal: Cessation of population-based vaccination in Indigenous communities may have led to exacerbation of TB incidence in these populations (e.g. Greenland)
[8], 2004	USA	QE	Native American (and Alaskan) Indigenous population			BCG vaccinated	Placebo	1483	1309	66/1309 (138 / 100,000 py)	36/1483 (66/100000 py)	52 (27–69)	Indigenous populations	Vaccine efficacy persisted for 50–60 years, suggesting long-term utility of vaccination
[30], 2001	USA	M	Homeless individuals			Vaccination	No vaccination	2 million	NA				Homeless population	Vaccination resulted in a 15.4% decline in TB cases among the chronically homeless and a 21.5% decline among the transiently homeless. Vaccination of 10% of the chronically homeless population led to a 10% decrease in TB cases and 2.4% decrease in TB-associated deaths over 10 years. Targeted BCG vaccination among the homeless HIV negative population is most beneficial if treatment for LTBI is available to those who have been vaccinated

*Can* Canada, *CC* Case-Control, *Cont* Control, *CS* Cross-Sectional, *CTR* Country, *Czh* Czech Republic, *Den* Denmark, *Fin* Finland, *Fr* France, *Grn* Greenland, *Inc* Incidence, *Int* Intervention, *Ire* Ireland, *IRR* Incidence Rate Ratio, *LI(I)C* Low (and Intermediate) Incidence Countries, *M* Modelling Study, *Nor* Norway, *PC* Prospective Cohort, *Pop* Population, *Py* Person years, *QE* Quasi-Experimental, *RC* Retrospective Cohort, *ST* Study Type, *Swe* Sweden, *UK* United Kingdom, *USA* United States of America

^a^ At the time of the study

^b^ Or case group in case-control studies

### BCG-associated adverse events and non-specific effects

Primary studies reporting adverse events are shown in Table 4. Of these, one reported BCG-associated osteomyelitis [10], three reported BCGitis [10, 44, 63] (including one case study in a French 4-month old [63], not shown in table), and 3 reported lymphadenitis [9, 37, 41]. Although the occurrence of serious adverse events as a result of vaccination was generally rare across studies, it was found that the use of unregistered vaccine strains was associated with a higher incidence of adverse events in comparison to registered vaccines, suggesting that particularly during vaccine shortages, when the implementation of unregistered vaccines is increased, improved surveillance and management of possible adverse events may be needed [37, 38].

**Table 4:** Secondary Outcomes: Adverse Events and other Outcomes under the Evaluated Prior and Current Vaccination Policies, by Country

Study Characteristics										Study Outcomes			Policy Recommendations	
Ref, Year	CTR	ST	Study Pop	Current / pior BCG policy (cov) ^a^	New policy (cov) ^a^	Int description ^b^	Cont description	N (int)	N (cont)	BCG-related adverse events			Target population / risk group	General Recommendations
										Event or Measure	Inc cont n/N	Inc Int n/N		
[37], 2016	Aus	CS	Australian children aged < 7 years	Targeted Vaccination (see risk groups)		Unregistered vaccine	Registered vaccine			Any AE (most commonly reported AEs: abscess (31%), injection site reaction (27%), lymphadenopathy/ lymphadenitis (17%)	87/100000 in 2009	201/100000 in 2014	Children < 5 at high risk (traveling to high-incidence countries (annual incidence 40 or more / 100,000) and aboriginal people	Surveillance of AEs during the use of unregistered vaccines due to shortages is important. Use of unregistered products may result in increased AEs.
[38], 2017	Aus	CS	Australian Children	Targeted Vaccination (see risk groups)		Unregistered vaccine (2012 onwards)	Registered vaccine (2001)	11,145 doses	8740 doses	Any AE	2/8740 (rate per 100,000 doses: 22.9 (0–54.6))	20/11145 (rate per 100,000 doses: 179.5 (100.9–258.0)		Need to monitor AEs, particular when unregistered vaccines are used.
[10], 1993	Czh	QE	Czech children	Until 1986, mass vaccination of infants born in a particular region of the country (in other regions, mass vaccination continues)		Region of ceased vaccination	Regions of continued mass vaccination	148,560	600,195	Osteomyelitis BCGitis		8 cases, 2 cases		(See Table 3)
[40], 2013	Den	CC	Danish general population	Routine BCG vaccination	Routine BCG vaccination ceased in 1985	With IBD	No IBD	474	5672	IBD (CD or UC)		Hazard ratio: 0.95 (95%CI: 0.75–1.1)		There was no significant effect of BCG on IBD overall (*p* = 0.14) (although being vaccinated with BCG early, before 4 months of age, was associated with a lower risk of IBD than not being vaccinated: HR =0.44 (95% CI, 0.21–0.96). BCG is safe for continued use regarding risk of IBD.
[59], 2016	Den	RCT	Infants (from birth to 13 months)	No routine vaccination of newborns		BCG vaccination within 7 days of birth	No BCG	2129	2133	Parent-reported early childhood infections	336/2099	291/2113 (IRR = 0.87 (95% confidence interval (CI): 0.72 to 1.05))		No significant non-specific public health benefit of vaccination (in terms of preventing non-TB infections)
[39], 2016b	Den	RCT	Infants	No routine vaccination of newborns		BCG vaccination within 7 days of birth	No BCG	1779	1674	Effect on child psychomotor development (Ages and Stages Questionnaire mean Score (SD))	179.9 (53.6)	178.2 (52.4) mean difference: −0.7 (BCG vs. control, 95%CI-3.7 to 2.4), *p* = 0.67		No significant negative effects of BCG on psychomotor development in infants was found
[41], 2016	Den	RCT	Newborns in Denmark	No routine vaccination of newborns		BCG Vaccination within 7 days of birth	No BCG	2118	NR	Supparative LA Regional LA		10 cases 13 cases		Few AEs and no fatalities linked to BCG
[60], 2010	Fr	O	French children admitted to the emergency department	Mass vaccination of newborns (or children before entry into daycare)	Targeted vaccination in high-risk groups at birth or within the first month of life (116/157 (73.9%))			Total: 224					Children: - Born in TB endemic countries or with at least one parent from an endemic country, - Traveling to TB endemic countries, - Having familial TB cases or being in contact with TB cases, - Living in precarious or overcrowded housing - Of low-socioeconomic status, − Residing in the ile-de-France or Guyane regions	41 infants falling into at least one of the risk categories were not vaccinated under the new (targeted) vaccination program. This indicates that through targeted vaccination of high-risk groups only, a proportion of children at risk may be missed, and that therefore, the discontinuation of universal vaccination needs to be accompanied by the strengthening of prevention, surveillance, and screening efforts
[14], 2014	Ne	CS	Dutch Children										- Newborn children with a parent born in a TB endemic country (TB incidence > 50 per 100,000 population. 2) - Immigrant children < 12 years, with no evidence of prior vaccination	39% of TB patients eligible for targeted vaccination (see target groups) had not been vaccinated, suggesting that further cases could have been prevented through stricter adherence to vaccination of high-risk children. Specifically, the continuation of targeted vaccination in new-borns of parents from TB endemic countries (WHO-estimated incidence > 50 /100.000 population) is recommended.
[61], 2008	Ne	RCT	Newborns with a family history of allergic disease (asthma, allergicrhinitis, eczema or food allergy)			BCG given at 6 weeks and repeated if BCG scar absent and TST negative at four months	Placebo	61	54	Asthma attack Medication use for eczema	8/54 (15%) 23/54 (43%)	11/61 (18%) RR: 1.22 (0.5–2.8) *p* value: ns 15/61 (25%) RR: 0.58 (0.3–1.0) *p* value = 0.04		BCG may be beneficial in reducing allergic disease, however, there was no significant difference in occurrence of asthma, although there was significantly lower medication use for eczema in the BCG group. Larger studies are needed however in order to confirm the effect of BCG on allergic disease.
[35], 2016	Nor	CS	Norwegian Children	Universal vaccination of children aged 13–15 until 2009	Targeted vaccination shortly after birth of children with at least one parent born in a country with high burden of TB (83.60%)			Total: 240,484					Children of parents from high-burden countries.	This study found that coverage of BCG vaccination, which was targeted, was lower than coverage of vaccines that are offered under a universal vaccination program, and suggests that improvements in the identification of children eligible for vaccination is needed, including the appropriate informing of healthcare professionals regarding the new guidelines.
[9], 2014	S. Ar	CS	Children diagnosed with BCG lymphadenitis	Mandatory BCG vaccination since 1968, mainly using Pasteur 1173 P2 and Tokyo 172–1. (98%)	Introduction of Danish 1331 strain in 2005					BCG-associated Lymphadenitis		42 cases, 41 of which received the Danish strain (and 1 the Tokyo strain).	NR	More comprehensive population-based studies before the introduction of a new vaccine strain, with particular attention to the prevalence of host risk factors that contraindicate vaccination, such as immunodeficiency.
[44], 1993	Swe	RC	Children < 6 years of age who were vaccinated	Vaccination of high-risk infants at birth (following cessation of routine vaccination in 1975)	Vaccination of high-risk infants at six months of age (7% (1979 to 1983),14% (1984 to 1990))			Total: 139000		Any AE Of these, the most common AE was Lymph node abscess: Disseminated BCG infection	268/139000 (I .9 per 1000 vaccinated children) 115/139000 4/139000 (3 of which had SCID).		High-risk infants	Targeted vaccination of high-risk groups should continue, however, the age should be moved from at birth to 6 months of age, to allow the detection of immune-compromising conditions prior to vaccination, to avoid serious AEs.
[62], 1995	Swe	RC	Children under 15	Routine vaccination of newborns until 1975 (95%)	Targeted vaccination of high-risk groups (post-1975) (2% prior t0 1980. Between 10 and 15% following 1980)	BCG vaccinated	BCG non-vaccinated			Atypical mycobacterial disease (incidence in children younger than 5)	26.8 per 100,000	4.6 per 100,000	High-risk infants	BCG vaccination may protect against atypical mycobacterial disease
[28], 1990	UAE	CS	Full term infants vaccinated at birth	Routine vaccination at birth				Total: 387		Sterile Abscesses at vaccination site (without lymphadenopathy)		3/387		Current vaccine is safe.

*AE* Adverse Event, *Aus* Australia, *Can* Canada, *CC* Case-Cohort, *CD* Crohn’s Disease, *Cont* Control, *CS* Cross-Sectional, *CTR* Country, *Czh* Czech Republic, *Den* Denmark, *Fr* France, *IBD* Inflammatory bowel disease, *Inc* Incidence, *Int* Intervention, *IRR* Incidence Rate Ratio, *LA* Lymphadenitis, *M* Modelling Study, *Ne* Netherlands, *Nor* Norway, *O* Observational Study, *PC* Prospective Cohort, *Pop* Population, *Py* Person years, *QE* Quasi-Experimental Study, *RC* Retrospective Cohort, *RCT* Randomized-controlled Trial, *S Ar* Saudi Arabia, *Swe* Sweden, *UAE* United Arab Emirates, *UC* Ulcerative Colitis, *UK* United Kingdom, *USA* United States of America

^a^ At the time of the study

^b^ Or case group in case-control studies

Regarding non-specific effects of the BCG vaccine, studies investigated its association with the development of inflammatory bowel disease (IBD) [40], child psychomotor development [39], and the incidence of childhood non-TB infections [59], allergic disease (such as asthma and eczema) [61], and atypical mycobacterial disease [62]. No significant association was found between BCG use and child psychomotor development [39] or incidence of IBD [40], non-TB infections [59], or asthma [61], whilst there was a lower incidence of atypical mycobacterial disease [62] and lower use of medication for eczema [61] in groups receiving BCG compared to those who did not.

### General recommendations in reviews and policy reports

A summary of recommendations from the 12 included policy reports is provided in Table 5**.** In general, mass vaccination was not recommended in low-incidence countries, where instead, TB control strategy should focus on the identification and rapid treatment of active cases as well as the control of LTBI [29]. In addition, it was emphasized in multiple reports that readiness for withdrawal necessitates a strong TB surveillance system to allow timely assessment of policy consequences [6, 19, 32], and comprehensive coverage of the selected high-risk groups should be ensured [65]. Re-vaccination was not recommended in any report, due to the lack of evidence for its efficacy [6, 32, 36, 65].

**Table 5:** Summary of Policy Reports

Study Characteristics						Reported Adverse Events			Recommendations	
Ref, Year	CTR	National TB incidence (at the time of study)	Study Pop	Current / prior BCG policy	New policy	Event	Inc Cont n/N	Inc Int n/N	Target population / risk group	Observations and General Recommendations
[19], 2006	Canada		Canadian Indigenous communities	Vaccination remains in some Indigenous communities only (discontinued in others since 2004)		Disseminated BCG infection	7 cases from 1993 to 2003 (6 fatal) in First Nations communities across Canada (all had an underlying immunodeficiency)		Communities with: - Average annual rate of smear positive pulmonary TB > 15/100000 in previous 3 years, or - Annual risk of TB infection > 0.1%, - Average annual notification rate of paediatric TB meningitis is > 1/10 million in children under 5 in the last 5 years	Should not be withdrawn in communities who continue to have high TB incidence. Consider withdrawal for low-risk communities. Readiness for withdrawal requires an effective surveillance system for monitoring of subsequent incident cases. Implications of withdrawal: As of 2006, 5 communities in the Sioux Look out zone met the criteria for high-risk (see left), and would thus be at risk for further TB transmission if BCG is withdrawn.
[64], 1994	UK	NR	General UK population	Routine vaccination in school programs					- Healthcare and prison staff, - Children of immigrants from high-prevalence countries, - Child or young adult contacts of active TB cases identified via contact tracing, - Babies of mothers with pulmonary TB	Routine vaccination of 10–14 year olds in schools (policy to be reviewed in 1995. In addition, vaccination of healthcare and prison staff, children of immigrants from high-prevalence countries, and child or young adult contacts of active TB cases. Babies of mothers with pulmonary TB should be vaccinated in the case of a negative TST six weeks after chemoprophylaxis.
[29], 1995	USA		US General population	NR						Routine vaccination is not recommended in the US. The primary strategy for TB control focuses on the identification and treatment of active cases, and the second priority is the identification of LTBI. BCG Vaccination should be considered among children with negative TSTs if they are in contact with an active TB case.
[65], 2006	France			Routine vaccination before entry into day-care	Selective vaccination of high-risk groups begun in 2004	BCGitis	Approx. 12 cases in total in France per year			At 50% efficacy for preventing all forms of TB in children under 15, 320 additional cases of childhood TB would occur if routine vaccination is stopped, however, 240 of these can be prevented by selective vaccination of only 15% of the child population, which would therefore also prevent 85% of BCG associated adverse events, and 11 of the 12 cases of BCGitis per year. Selective vaccination is therefore recommended, but caution is advised to ensure appropriate coverage of target populations. Revaccination: Not recommended (no evidence of efficacy)
[66], 2012	Australia	5.28 and 5.95 cases per 100,000 population in 2005–2009	General population of Australia	Selective vaccination of high-risk groups					- Aboriginal and Torres Strait Islander neonates in communities with a high incidence of TB; - Neonates and children 5 years of age and under who will be travelling to or living in countries or areas with a high prevalence of TB for extended periods; - Neonates born to parents with leprosy or a family history of leprosy.	Most TB cases (80–90%) are among immigrants from high-prevalence countries. Selective vaccination is therefore used. Vaccination is recommended for:1. Aboriginal and Torres Strait Islander neonates in communities with a high incidence of TB;2. Neonates and children 5 years of age and under who will be travelling to or living in countries or areas with a high prevalence of TB for extended periods;3. Neonates born to parents with leprosy or a family history of leprosy. BCG vaccination may be considered in Children over 5 years of age who will be travelling to or living in countries or areas with a high prevalenceof TB for extended periods; Revaccination: Not recommended
[47], 1996	UK		High-incidence communities in the UK	NR						Selective vaccination in high-incidence communities (over 40 cases per 100,000) as well as of immigrants from endemic areas may be of interest, although this is more difficult to implement effectively, as a universal vaccination program would reduce the number of eligible people missed
[33], 2011	Canada	4.8/100,000	Canadian First nations population	Routine vaccination discontinued in Alberta, Saskatchewan, Quebec and some Ontario First Nations communities (Moose Factory and Thunder Bay Zones) between 2003 and 2005.		Disseminated BCG infection	6 cases between 1993 and 2003 (estimated rate: 205 cases per million doses)		- Infants in First Nations and Inuit communities or infants in communities with an average annual rate of smear-positive pulmonary TB greater than 15 per 100,000 population in the past 3 years, or - Infants residing in populations with an annual risk of TB infection greater than 0.1%, (if early identification and treatment of TB infection are not available.)	The 2011 Report found no increase in TB meningitis or miliary TB since the discontinuation of routine vaccination. However, due to the small population sizes of first nations communities, incidence rates easily fluctuate above or below the National Advisory Committee on Immunisation (NACI) cut-offs (see left) and therefore perhaps make these of limited use in determining appropriate vaccination policy.
[45], 2014	Egypt	34 cases to 17 cases per 100,000 population (1992–2011)	General Egyptian population	Routine vaccination at birth (1992: 92%, 2006: 99%)						There was a significant correlation between vaccine coverage and TB incidence, prevalence, TB-associated mortality, and TB associated under five mortality in 1992–2011: (R, *p* value): 0.74 < 0.0001, 0.86 < 0.0001, 0.86 < 0.0001, 0.77 < 0.0001. Current routine vaccination therefore remains effective.
[31], 2006	Finland	6.6/100000 in 2004	General Finnish population	Routine vaccination at birth (98%)	Targeted vaccination among high-risk groups (planned to begin 2008)	BCG osteitis	2 cases in 2002	6 cases in 2003	- Children of immigrant families from high-incidence countries - Children of Finnish-born parents with a family history of TB - Children of families planning to stay for a prolonged period in a high-incidence country - Upon request by the parent	In Finland, most TB cases are among seniors (65 or older) and the Indigenous population (rather than among immigrants, as is the case in many other European countries).
[46], 2007	UK			Vaccination of all secondary school children	Targeted BCG vaccination both in neonates and opportunistically in older children				- Infants attending primary care in areas with a high incidence of TB Infants who: - Were born in an area with a high incidence of tuberculosis, or - Have one or more parents or grandparents who were bornin a high-incidence country (> 40 cases per 100,000 per year), or - Have a family history of tuberculosis in the past 5 years. Children younger than 16 who also meet these risk criteria should be opportunistically vaccinated.	See target groups
[32], 1994	Finland		Finnish General population	NR (50–80%)						Necessities before discontinuation (International union against TB and lung disease): - Strong, established TB control program for TB case data over past 5 years, - Knowledge of the impact of HIV prevalence in the population on TB transmission - Incidence of smear-positive TB no more than 5 / 100,000 in past 3 years, or - < 1 per 10,000,000 cases of TB meningitis in children under 5 in the last 5 years, or - Annual risk of TB 0.1% or less. Implications of withdrawal: discontinuation of mass vaccination resulted in an increase of TB in Sweden1 and in former Czechoslovakia Revaccination: Not recommended
[6], 2001	Global	Varied		Varied						International Union against Tuberculosis and LungDisease recommends that a country should only move from universal to targeted vaccination if: - An efficient system of case notification is established- The average annual notification rate of smear-positive pulmonary tuberculosis is < 5 per 100,000;or - The average annual notification rate of TB meningitis in children aged < 5 years is < 1 per 10 million population over the previous 5 years, or the average annual risk of TB infection is < 0.1% Revaccination: Not recommended

*Cont* Control, *CTR* Country, *Inc* Incidence, *Int* Intervention, *NR* Not reported

Among the 5 included reviews, the 3 that made recommendations for general low-incidence settings all concluded that a universal vaccination policy is of limited value in low-incidence countries, with targeted vaccination among high-risk groups being recommended instead [5, 67, 68]. The 2 remaining reviews focused specifically on the UK and France, and were both published prior to the discontinuation of mass vaccination in both countries, with the French review arguing that mass vaccination at the time (2003) continued to be justified based on the number of incident TB meningitis cases, although a precise cut-off for the number of cases at which vaccination would no longer be considered beneficial was not provided [69], whilst the British study stated that (as of 1988), routine vaccination of 10–14 year olds within British school programs continued to be justified, but that this policy is subject to revision in future years [70]. As previously mentioned, mass vaccination policies in both countries have since been revised, in favour of the adoption of targeted vaccination [12, 13].

### Risk of Bias assessment

**Fig. 2:**
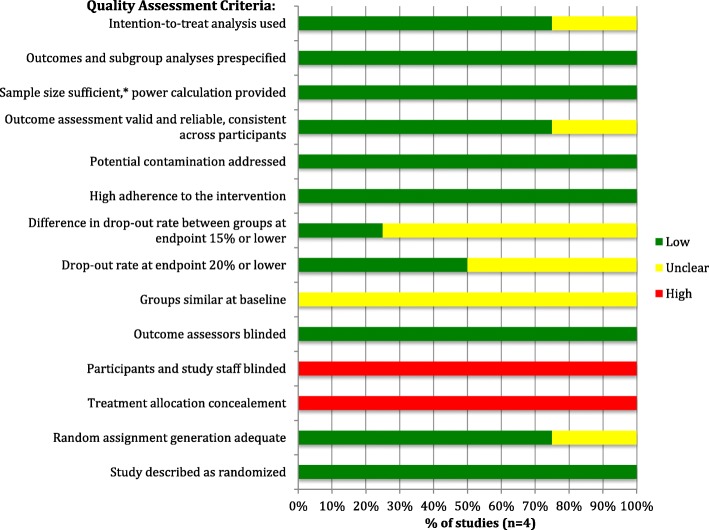
Risk of Bias Assessment for Included Randomized Controlled Trials. * Sample size sufficient to detect a between group difference in main outcome with at least 80% power.

The risk of bias in included RCTs (*n* = 4) was assessed using the National Heart, Lung and Blood Institute’s (NHLBI) Quality Assessment Tool for Controlled Intervention Studies [25]. The included RCTs were generally found to have a low risk of bias, as the risk of cross contamination (i.e. vaccination occurring in the non-vaccinated group) was low, sample sizes were justified, and group allocation was appropriately randomized in most studies. Potential sources of bias in the included RCTs however include the fact that, due to the nature of the intervention (i.e. administering the vaccination) study staff could not be blinded to group assignment, and group allocation could not be concealed from participants. Risk of bias assessment results for the included RCTs is shown in Fig. 2.

**Fig. 3:**
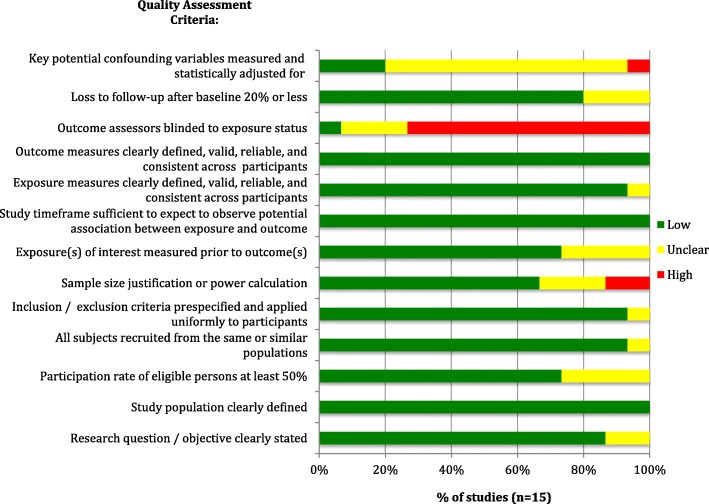
Risk of Bias Assessment for Included Observational Studies

**Fig. 4:**
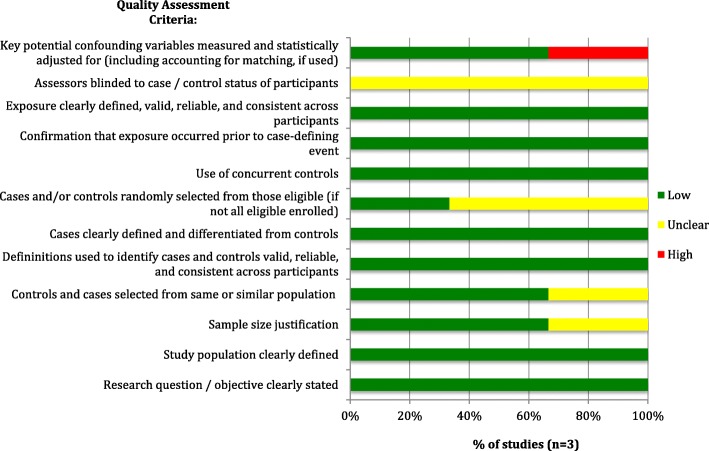
Risk of Bias Assessment for Included Case-Control Studies

Observational and cross-sectional studies (*n* = 15) (assessed via the NHLBI’s Quality Assessment Tool for Observational Cohort and Cross-Sectional Studies [25]) were also generally of high methodological quality, meeting most of the quality assessment criteria. However, lack of blinding of outcome assessors (or lack of reporting thereof) was a potential source of bias in most studies, and there was, in most studies, inadequate or unclear reporting of adjustment for confounders, as shown in Fig. 3 below. The included case-control and case-cohort studies (*n* = 3) (evaluated using the NHLBI’s Quality Assessment Tool for Case-control Studies [25]) also insufficiently reported blinding of outcome assessors to the status of participants (as cases or controls), although other sources of bias were appropriately addressed in all studies, such as providing clear case definitions and differentiating them clearly from controls. The risk of bias assessment for case-control studies is shown in Fig. 4**.**

**Fig. 5:**
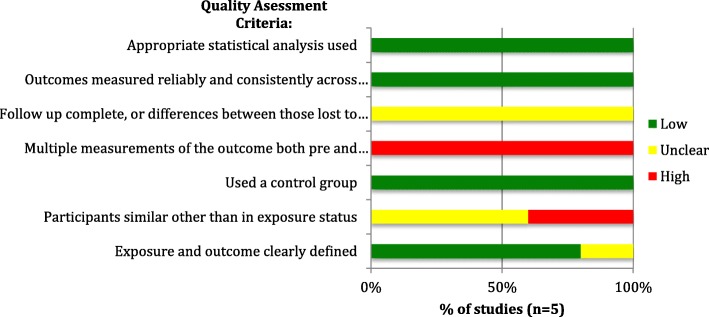
Risk of Bias Assessment for Included Quasi-Experimental Studies

In the case of quasi-experimental studies (*n* = 5), which were evaluated using the JBI Critical Appraisal Checklist for Quasi-Experimental Studies [26], considerable sources of bias were the fact that most studies did not provide sufficient information regarding participants’ baseline characteristics and whether there were any significant differences in these characteristics in the exposed vs. unexposed groups, as well as insufficient or no comparison between the characteristics of participants lost to follow-up compared to those who completed the study. Figure 5 displays the risk of bias assessment for the included quasi-experimental studies, and individual study scores for each quality assessment criterion in Figs. 2, 3, 4, 5 are provided in Additional file 2.

The case report [63] was evaluated using the JBI Critical Appraisal Checklist for Case Reports [26], and was found to have a low risk of bias, as it clearly described the patient’s demographic characteristics and clinical history, as well as the patient’s current condition, the administered diagnostic tests and the treatment outcome.

Lastly, the 4 included modelling studies were assessed using the ISPOR Principles of Good Practice for Decision Analytic Modelling in Health-Care Evaluation [27]. All modelling studies used a model structure that was consistent with the nature of the disease, and in the case of Markov models (*n* = 2), health states relevant to the disease were included and the probabilities of transition between these states took into account patient history and treatment. However, most did not clearly state that their model inputs were derived from a comprehensive review of the literature, and only 2 conducted sensitivity analyses [30, 51]. In addition, none provided information on whether or where the source code for the model is accessible for peer-review.

## Discussion

### Summary of evidence

Given the variation in its reported efficacy and the high number of vaccinations needed to prevent one TB case in a low-incidence setting, there was general consensus among the reviewed studies that the replacement of universal screening with targeted screening of high-risk groups is justified in low-burden countries [5, 10, 11, 67, 68]. Such targeted screening programs however require establishing precise guidelines or incidence cut-offs on which to base the categorization of high-risk groups. In this regard, it is relevant to note that a recent study among Canadian, Alaskan and Greenland Indigenous populations found that population-based vaccination of infants, along with screening and treatment for LTBI, was significantly associated with a decrease in TB incidence [55], which suggests that population-level vaccination in high-risk communities as a whole, rather than vaccination based on individual risk factors, may be beneficial in the Canadian Indigenous community context. In terms of the strength of this evidence however, any causal inferences should be made with caution in the case of population-based studies such as this, given the uncertainty in individual exposure. The benefit of population-level vaccination however is supported by findings of a simulation study of optimal BCG vaccination strategies among children in low-incidence countries, which concluded that community-wide vaccination is beneficial in scenarios of communities with a prevalence of approximately 30 smear-positive TB cases / 100,000 [52]. The strength of this evidence is judged to be considerable, given that this modelling study adhered to most of the quality assessment criteria in the aforementioned ISPOR guidelines for decision-analytic models (see Table 6). Therefore, as the Canadian Indigenous population has a similar incidence of TB (23.5/100,000) [18], community-level vaccination may be beneficial in this setting.

**Table 6:** Risk of Bias Assessment for Modeling Studies

Criteria ^a^	Criteria met (yes = 1, no/unclear = 0, NA = not applicable)			
	Study (Author, Year)			
	Manissero, 2008	Sutherland, 1989	Brewer, 2001	Gerberry, 2009
Model Structure:				
Inputs and outputs relevant to decision-making	1	1	1	1
Structure consistent with the theory of the disease	1	1	1	1
Structure is simple but includes essential elements for vaccine policy decision-making (and any simplifications are justified)	1	1	1	1
Heterogeneity in the modelled population accounted for by stratifying by groups that have different outcome probabilities or costs	1	0	1	1
Time horizon of the model sufficient to detect important (and clinically meaningful) differences in long-term health and cost outcomes	0	1	1	0
For Markov models / health state transition models:				
Health states appropriately defined, relevant subdivisions of health states included	NA	NA	1	1
Where relevant, transition probabilities take into account clinical history	NA	NA	1	1
Choice of cycle length justified	NA	NA	0	0
Data Identification:				
Systematic reviews of the literature conducted on key model inputs	0	0	1	0
Ranges provided in base-case estimates of all input parameters for which sensitivity analyses were done	NA	NA	1	1
Data based on expert opinion, if used, are derived via formal methods, e.g. Delphi	NA	NA	NA	NA
Attempts to obtain new data prior to modeling have been considered	1	1	1	1
Data Incorporation and Modeling:				
Modeling methods follow accepted methods of biostatistics and epidemiology	1	1	1	1
Included sensitivity analyses of key parameters	0	0	1	1
Model validation:				
Model subjected to internal testing through input of extreme values (or equal values for replication testing)	1	0	0	1
Calibration data, where available, should be from sources independent of those used to estimate inputs	1	0	0	1
Source code available for peer-review	0	0	0	0
Model based on the best evidence available at the time	1	1	1	1

^a^ Criteria based on the ISPOR Principles of Good Practice for Decision Analytic Modelling in Health-Care Evaluation [27]

Given the findings of these two studies, it may therefore be of interest to consider community-level incidence as an indicator of the potential utility of targeted BCG vaccination in any particular Indigenous community. With this in mind, according to the Canadian Immunization Guidelines (based on those by the International Union Against TB and Lung Disease [71]), although routine vaccination is not recommended in any Canadian population, risk groups in which vaccination can be considered include infants in First Nations or Inuit communities with an average annual incidence of smear-positive pulmonary TB > 15 per 100,000 population over the past 3 years, or infants in communities with an annual risk of TB infection > 0.1% [70]. .Unfortunately however, as noted by a previous policy report on BCG vaccination in Canadian Indigenous communities, due to the small population sizes of First Nations communities, incidence rates easily fluctuate above or below specific incidence cut-offs provided in vaccination guidelines, and therefore may make these of limited use in determining appropriate vaccination policy [33]. In addition, it is relevant to note that re-introduction of BCG vaccination in a given community will limit the value of the TST as a useful screening tool for LTBI [10], which is especially relevant in the Canadian indigenous setting, where screening for LTBI remains a component of the ongoing TB control strategy and access to interferon-γ-release assays is limited [58].

Additional considerations include that if routine BCG vaccination is ultimately re-introduced to Canadian Indigenous communities, based on the findings of studies in the current review, it is recommended that the age of vaccination be increased from at birth to 6 months of age, given that higher protective efficacy has been shown with vaccination at 6 months compared to before 6 months of age (63% vs. 42% efficacy, respectively), (in a study with moderate strength of evidence due to a small sample size, but otherwise having low risk of bias) [48] and that vaccination at 6 months rather than at birth allows sufficient time for the identification of any potential underlying immune-deficiencies that could result in disseminated BCGitis following vaccination (in a study with a large sample size (*n* = 139,000) and generally low risk of bias (see Fig. 3) [44]. This decision would however need to be considered in light of the logistical challenges of conducting TST testing prior to offering BCG at 6 months, given the need for follow-up 48 to 72 h after TST administration.

### Limitations

The heterogeneity of the included studies, in terms of study design, study populations, and outcome measures, represents a limitation of this review, given that it precluded the conduct of a meta-analysis. In addition, although included studies generally had a low risk of bias, due to the overt nature of the intervention (receiving the BCG vaccine), many primary studies could not take measures to blind outcome assessors to participants’ intervention assignment (vaccinated or non-vaccinated). A further limitation of this review and the resulting policy recommendations includes that although the findings of the included studies are potentially relevant to the Canadian Indigenous community context in so far as they focus on high-risk populations in low-incidence settings, Canadian Indigenous communities represent a unique setting with additional challenges including their remoteness and vast, sparsely-populated areas. These unique characteristics therefore may make some of the findings regarding optimal vaccination policies in other high-risk groups in low-incidence countries less applicable to the Canadian Indigenous context (for example the aforementioned limited applicability of incidence-based vaccination policies in the context of small populations with easily fluctuating incidence rates).

## Conclusions

Firstly considering the risk-benefit of implementing BCG vaccination, in terms of vaccine safety, given the generally low incidence of serious adverse events associated with BCG vaccination reported by the included studies, the vaccine can be considered generally safe for use in immune-competent hosts. The reported protective efficacy of the BCG vaccine against tuberculosis however varies widely [50, 53].

In this context of relatively low risk and potential benefit, BCG vaccination in Canadian Indigenous communities and epidemiologically similar high-risk groups in low-incidence countries may be considered. In the Canadian Indigenous setting in particular, a community-level vaccination approach could be considered [55], targeting communities with an incidence of smear-positive pulmonary TB > 15 / 100,000, or an annual risk of TB > 0.1% [72]. However, the limited utility of precise incidence cut-offs for targeted vaccination in sparsely-populated communities (such as Canadian Indigenous communities), in which a small number of cases can cause considerable fluctuations in community-level incidence must be noted [33].

### Implications of BCG vaccine implementation

Where BCG vaccination is implemented, delivery strategies and potential barriers to achieving adequate coverage should be considered, which is particularly relevant in Canadian Indigenous communities and other hard-to-reach settings. Furthermore, given that vaccination at birth has been shown not to allow sufficient time to identify possible immune-deficiencies in infants, consequently putting them at risk for disseminated BCGitis, increasing the age at which BCG is administered from at birth to at 6 months of age should be considered (although keeping in mind the logistical challenge of then needing to administer a TST prior to vaccination at 6 months) [19, 44]. In addition, in the context of the varied history of BCG vaccination in Canada and other low-incidence countries, and potentially ongoing differences in implementation across communities, education of healthcare providers regarding the interpretation of positive TSTs is needed in order to ensure continued adequate identification and subsequent treatment of LTBI.

### Implications of withdrawal

The withdrawal of universal BCG vaccination, even when replaced with a targeted vaccination policy, may have adverse implications, including a potential increase in TB incidence. This is particularly true if the high-risk groups recommended for vaccination under a targeted program are not effectively identified and subsequently vaccinated. This implication is particularly alarming considering that 3 studies reported subsequent incomplete vaccine coverage of high-risk groups following withdrawal of mass vaccination [14, 35, 60], (Table 4) highlighting potential negative implications of discontinuation of universal programs, and the need to improve strategies to identify and reach the selected at-risk groups when shifting to a targeted vaccination policy. Therefore, in cases where an existing vaccination program is discontinued, it is imperative that an effective TB surveillance system is in place and well established before the withdrawal of the program, and that case-finding, screening, and diagnostic efforts are strengthened in order to ensure continued TB control [19, 20]. This is particularly relevant to highlight in reference to the Canadian Indigenous setting, where significant barriers persist regarding surveillance, screening, and diagnostic efforts in remote and under-served communities.

## Supplementary information

**Additional file 1.** Systematic Review Search Strategy. A detailed outline of the search strategy used in this systematic review, including the number of records returned for each search term.

**Additional file 2.** Individual Study Quality Assessment Scores for RCTs, Observational, Case-control and Quasi-experimental studies. A table providing quality assessment scores (low, unclear or high) for each criterion assessed for included RCTs, observational, case-control and quasi-experimental studies.

## Data Availability

All data collected during this review are contained and referenced within this manuscript and its additional files.

## Abbreviations

BCG: Bacille Calmette-Guérin
HIV: Human Immunodeficiency Virus
LTBI: Latent Tuberculosis Infection
TB: Tuberculosis
TST: Tuberculin Skin Test

## Footnotes

1 TB incidence in Greenland as of 2016 is > 100 per million population, but this study was nonetheless included given its information on vaccination in Canadian and Alaskan Indigenous communities
