# Supplementary material for: A systematic review of BCG vaccination policies among high-risk groups in low TB-burden countries: implications for vaccination strategy in Canadian indigenous communities
Source: BMC Public Health. 2019 Nov 11;19:1504. doi: 10.1186/s12889-019-7868-9 (PMC6849173; doi:10.1186/s12889-019-7868-9)
Supplement: Supplementary file 1 — Additional file 1. Systematic Review Search Strategy. A detailed outline of the search strategy used in this systematic review, including the number of records returned for each search term. [file 12889_2019_7868_MOESM1_ESM.docx]

**Additional file 1**

**Systematic Review Search Strategy**

Search Date: Jan 19^th^, 2018

Database: Medline (OVID)

| Step | Search Term | Records |
| --- | --- | --- |
| 1 | BCG Vaccine/ | 19536 |
| 2 | Bacillus Calmette Guerin.mp. | 5942 |
| 3 | Bacille Calmette Guerin.mp. | 2504 |
| 4 | bcg.mp. | 29633 |
| 5 | Tuberculosis/ | 103417 |
| 6 | tuberculosis.mp. | 245910 |
| 7 | tb.mp. | 45018 |
| 8 | 5 or 6 or 7 | 257369 |
| 9 | 1 or 2 or 3 or 4 | 30940 |
| 10 | (Angola or Bangladesh or Brazil or Cambodia or China or Congo or Central African Republic or (DPR Korea or North Korea or DPRK) or Ethiopia or India or Indonesia or Kenya or Lesotho or Liberia or Mozambique or Myanmar or Namibia or Nigeria or Pakistan or Papua New Guinea or Philippines or Russian Federation or Sierra Leone or South Africa or Thailand or Tanzania or Viet Nam or Vietnam or Zambia or Zimbabwe).mp. [mp=title, abstract, original title, name of substance word, subject heading word, keyword heading word, protocol supplementary concept word, rare disease supplementary concept word, unique identifier, synonyms] | 702318 |
| 11 | Public Health/ or Health Policy/ | 135211 |
| 12 | Public Health Practice/ | 5358 |
| 13 | Vaccination/ | 77462 |
| 14 | (vaccination practice* or vaccination polic*).mp. [mp=title, abstract, original title, name of substance word, subject heading word, keyword heading word, protocol supplementary concept word, rare disease supplementary concept word, unique identifier, synonyms] | 1491 |
| 15 | (health polic* or health practice*).mp. [mp=title, abstract, original title, name of substance word, subject heading word, keyword heading word, protocol supplementary concept word, rare disease supplementary concept word, unique identifier, synonyms] | 90183 |
| 16 | 11 or 12 or 13 or 14 or 15 | 236196 |
| 17 | 8 and 9 and 16 | 2916 |
| 18 | 17 not 10 | 2729 |
| 19 | limit 18 to (yr="1988 -Current" and (english or french)) | 1049 |

Database: Embase (OVID)

|  |
| --- |

| Step | Search Term | Records |
| --- | --- | --- |
| 1 | BCG Vaccine/ | 42644 |
| 2 | (Bacillus Calmette Guerin or Bacille Calmette Guerin or bcg).mp. [mp=title, abstract, heading word, drug trade name, original title, device manufacturer, drug manufacturer, device trade name, keyword, floating subheading word] | 56862 |
| 3 | tuberculosis/ | 141097 |
| 4 | (tuberculosis or tb).mp. [mp=title, abstract, heading word, drug trade name, original title, device manufacturer, drug manufacturer, device trade name, keyword, floating subheading word] | 308997 |
| 5 | vaccination/ | 137439 |
| 6 | (Angola or Bangladesh or Brazil or Cambodia or China or Congo or Central African Republic or (DPR Korea or North Korea or DPRK) or Ethiopia or India or Indonesia or Kenya or Lesotho or Liberia or Mozambique or Myanmar or Namibia or Nigeria or Pakistan or Papua New Guinea or Philippines or Russian Federation or Sierra Leone or South Africa or Thailand or Tanzania or Viet Nam or Vietnam or Zambia or Zimbabwe).mp. [mp=title, abstract, heading word, drug trade name, original title, device manufacturer, drug manufacturer, device trade name, keyword, floating subheading word] | 867572 |
| 7 | health care policy/ | 173030 |
| 8 | (polic* or practice*).mp. [mp=title, abstract, heading word, drug trade name, original title, device manufacturer, drug manufacturer, device trade name, keyword, floating subheading word] | 1785667 |
| 9 | 1 or 2 | 56862 |
| 10 | 3 or 4 | 309002 |
| 11 | 7 or 8 | 1785673 |
| 12 | 5 and 9 and 10 and 11 | 314 |
| 13 | 12 not 6 | 274 |
| 14 | limit 13 to (yr="1988 -Current" and (english or french)) | 194 |
