# Supplementary material for: A systematic review of BCG vaccination policies among high-risk groups in low TB-burden countries: implications for vaccination strategy in Canadian indigenous communities
Source: BMC Public Health. 2019 Nov 11;19:1504. doi: 10.1186/s12889-019-7868-9 (PMC6849173; doi:10.1186/s12889-019-7868-9)
Supplement: Supplementary file 2 — Additional file 2. Individual Study Quality Assessment Scores for RCTs, Observational, Case-control and Quasi-experimental studies. A table providing quality assessment scores (low, unclear or high) for each criterion assessed for included RCTs, observational, case-control and quasi-experimental studies. [file 12889_2019_7868_MOESM2_ESM.docx]

**Additional File 2:** Individual Study Quality Assessment Scores for RCTs, Observational, Case-control and Quasi-experimental studies

| Study (Author, Year) | Risk of Bias Assessment Criteria and Level of Risk of Bias (low, unclear, high) | | | | | | | | | | | | | |
| --- | --- | --- | --- | --- | --- | --- | --- | --- | --- | --- | --- | --- | --- | --- |
|  | Randomized Controlled Trials | | | | | | | | | | | | | |
|  | Study was randomized, a randomized trial, a randomized clinical trial, or an RCT? | Method of randomization adequate? (random assignment generation) | Treatment allocation concealment | Participants and study staff blinded | Outcome assessors blinded | Groups similar at baseline (on relevant demographics) | Drop-out rate at endpoint 20% or lower (of those allocated to treatment) | Difference in drop-out rate between groups at endpoint 15% or lower | High adherence to the intervention | Other interventions avoided or similar between groups (contamination) | Outcome assessment valid and reliable, consistent across participants | Sample size sufficient to detect a between group difference in main outcome with at least 80% power | Outcomes and subgroup analyses prespecified | Intention-to-treat analysis used (analysis according to original group assignment) |
| Kjaergaard, 2016 | low | low | high | high | low | unclear | unclear | unclear | low | low | low | low | low | low |
| Kjaergaard, 2016b | low | low | high | high | low | unclear | unclear | unclear | low | low | unclear | low | low | low |
| Nissen, 2016 | low | low | high | high | low | unclear | low | unclear | low | low | low | low | low | low |
| Steenhuis, 2008 | low | unclear | high | high | low | unclear | low | low | low | low | low | low | low | unclear |
| Observational Studies | | | | | | | | | | | | | | |
|  | Research question / objective clearly stated | Study population clearly defined | Participation rate of eligible persons at least 50% | All subjects recruited from the same or similar population | Inclusion / exclusion criteria pre-specified and applied uniformly to all participants | Sample size justification or power calculation | Exposure(s) of interest measured prior to outcome(s) | Study timeframe sufficient to expect to observe potential association between exposure and outcome | Exposure measures clearly defined, valid, reliable, and implemented consistently across study participants | Outcome measures clearly defined, valid, reliable, and implemented consistently across study participants | Outcome assessors blinded to exposure status | Loss to follow-up after baseline 20% or less | Key potential confounding variables measured and statistically adjusted for | |
| Al-Hajoj, 2014 | low | low | low | low | low | high | unclear | low | low | low | high | low | unclear | |
| Dankova, 1994 | unclear | low | unclear | low | low | high | low | low | low | low | high | unclear | unclear | |
| Danvin, 2010 | low | low | low | low | low | low | low | low | low | low | low | low | low | |
| Dehghani, 2018 | low | low | low | unclear | low | low | unclear | low | unclear | low | high | unclear | low | |
| Erkens, 2014 | low | low | low | low | low | low | low | low | low | low | unclear | low | low | |
| Feiring, 2016 | low | low | low | low | low | low | low | low | low | low | unclear | low | high | |
| Frankenberg, 1991 | unclear | low | low | low | low | low | low | low | low | low | high | low | unclear | |
| Hendry, 2016 | low | low | low | low | low | low | low | low | low | low | high | low | unclear | |
| Khandaker, 2017 | low | low | unclear | low | low | unclear | low | low | low | low | high | low | unclear | |
| Romanus, 1993 | low | low | low | low | low | low | low | low | low | low | high | low | unclear | |
| Romanus, 1995 | low | low | unclear | low | low | unclear | unclear | low | low | low | high | unclear | unclear | |
| Romanus, 2006 | low | low | low | low | low | low | low | low | low | low | high | low | unclear | |
| Schwoebel, 1994 | low | low | low | low | low | low | unclear | low | low | low | unclear | low | unclear | |
| Sedaghatian, 1990 | low | low | unclear | low | unclear | unclear | low | low | low | low | high | low | unclear | |
| Van Bui, 2015 | low | low | low | low | low | low | low | low | low | low | high | low | unclear | |
| Case-Control Studies | | | | | | | | | | | | | | |
|  | Research question / objective clearly stated | Study population clearly defined | Sample size justification | Controls and cases selected from same or similar population | Definitions used to identify cases and controls valid, reliable, and consistent across participants | Cases clearly defined and differentiated from controls | Cases and/or controls randomly selected from those eligible (if not all eligible enrolled) | Use of concurrent controls | Confirmation that exposure occurred prior to case-defining event | Exposure clearly defined, valid, reliable, and consistent across participants | Assessors blinded to case / control status of participants | Key potential confounding variables measured and statistically adjusted for (including accounting for matching, if used) | | |
| Houston, 1990 | low | low | low | unclear | low | low | unclear | low | low | low | unclear | high | | |
| Rodrigues, 1991 | low | low | unclear | low | low | low | unclear | low | low | low | unclear | low | | |
| Villumsen, 2013 | low | low | low | low | low | low | low | low | low | low | unclear | low | | |
| Quasi-Experimental Studies | | | | | | | | | | | | | | |
|  | Exposure and outcome clearly defined | Participants similar other than in exposure status | Used a control group | Multiple measurements of the outcome both pre and post exposure | Follow up complete, and if not, differences between those lost to follow up and those retained adequately analyzed | Outcomes measured reliably and consistently across participants | Appropriate statistical analysis used |  |  |  |  |  |  |  |
| Aronson, 2004 | low | unclear | low | high | unclear | low | low |  |  |  |  |  |  |  |
| Brantsaeter, 2009 | low | high | low | high | unclear | low | low |  |  |  |  |  |  |  |
| Che, 2011 | unclear | unclear | low | high | unclear | low | low |  |  |  |  |  |  |  |
| Kelly, 1997 | low | high | low | high | unclear | low | low |  |  |  |  |  |  |  |
| Trnka, 1993 | low | unclear | low | high | unclear | low | low |  |  |  |  |  |  |  |
